# Cell death responses to acute high light mediated by non-photochemical quenching in the dinoflagellate *Karenia brevis*

**DOI:** 10.1038/s41598-022-18056-4

**Published:** 2022-08-18

**Authors:** Yida Gao, Deana L. Erdner

**Affiliations:** 1 University of Texas at Austin, Marine Science Institute, Port Aransas, TX 78373 USA; 2 Present Address: Florida Fish and Wildlife Conservation Commission, Fish and Wildlife Research Institute, St. Petersburg, FL 33701 USA

**Keywords:** Microbiology, Ecology

## Abstract

Programmed cell death (PCD) can be induced in microalgae by many abiotic challenges via generation of reactive oxygen species (ROS). Marine phytoplankton live in a highly variable light environment, yet the potential for excess photosynthetically available radiation to trigger PCD has not been examined. On the other hand, photoprotective non-photochemical quenching (NPQ) is hypothesized to counteract intracellular ROS, potentially preventing cell death. The main objective of this study is to investigate high-light-induced death processes and their relationship with photosynthesis in bloom-forming dinoflagellate *Karenia brevis*. Here, we characterized the prevalence of ROS, caspase-like enzyme activity and cell death as well as photosynthetic status under acute irradiance of 500, 750 or 1000 µmol m^−2^ s^−1^. PCD only occurred at the largest light shift. Although depressed photosynthetic capacities and oxidative stress were apparent across the stress gradient, they did not necessarily lead to cell death. NPQ exhibited dose-dependent activation with increasing light stress, which enabled cells to resist or delay PCD. These results highlight the important role of the balance between ROS generation and NPQ activation on determining cell fates in *Karenia* under acute irradiance stress. This research also provides insights into potential survival strategies and mechanisms of cell loss under a changeable light environment.

## Introduction

The dinoflagellate alga *Karenia brevis* is responsible for frequent harmful algal bloom (HAB) events in the Gulf of Mexico (GoM). They produce potent neurotoxins called brevetoxins, which can lead to animal death, human sickness, and significant loss in fishing and tourism industries^1^. The mechanisms of *K. brevis* bloom initiation have received far more attention than the causes of bloom declines^2–4^, which remain poorly understood. During the past two decades, the development of molecular and immunological tools has revealed the impact of programmed cell death (PCD) in causing lysis of many cosmopolitan algal species^5^. PCD refers to a variety of genetically controlled self-destruction pathways^5^, and it can be induced by biotic and abiotic challenges such as viral attack, CO_2_ limitation, and iron starvation, potentially leading to decline of algal blooms^6–9^.

Marine phytoplankton live in a highly variable light environment, yet the potential for excess photosynthetically available radiation (PAR) to trigger PCD in phytoplankton has not been investigated. Most algal cell-death research relating to irradiance has focused on ultraviolet (UV) exposure and dark deprivation^10–12^. However, high light can be damaging to cells, potentially to the point of inducing PCD. Absorption of light energy beyond photosynthetic capacity results in excessive excitation of electrons from chlorophyll^13,14^. These over-excited electrons would be transferred to O_2_ within the chloroplast leading to the generation of various reactive oxygen species (ROS)^15,16^. In the PCD cascade, ROS serves as an intracellular signal to activate caspase-like activity in phytoplankton cells and thus lead to cell death^17^. High light can induce photoinhibition in *K. brevis*^18–21^, which could increase ROS and trigger PCD. On the other hand, the intracellular accumulation of ROS can be counteracted by activation of photoprotective non-photochemical quenching (NPQ), which dissipates excessive excitation energy as heat^22^. However, under high light NPQ induction and its effects in *K. brevis* have not yet been described. Given the fact that *K. brevis* in the GoM may experience rapid and drastic variation in light by more than 700 µmol m^−2^ s^−1^ within minutes as a result of highly changeable weather and intense surface water mixing^20,23,24^, characterizing their responses to light can inform our understanding of bloom development and decline.

This study investigated photosynthetic and stress-related death processes in response to different levels of acute high light stress in *K. brevis*. We hypothesized that NPQ would be induced to mitigate ROS production and therefore prevent cell death under high light stress in *K. brevis*. Two strains of *K. brevis* maintained under irradiance of 50 µmol m^−2^ s^−1^ were transferred to 500, 750 or 1000 µmol m^−2^ s^−1^ for two light periods. We used stress and cell death markers (ROS, caspase-like enzyme activity and SYTOX) to characterize high-light-induced death responses. Since ROS production is closely linked to photoinhibition in dinoflagellates, photosynthetic status was characterized using NPQ, Fv/Fm, and rapid light curves (RLC). Our results show interrelationships between photosynthetic responses and PCD expression and highlight the important roles of ROS production and removal on determining cell fates under acute irradiance stress, thereby providing insights into how high light may affect maintenance and decline of *Karenia* blooms.

## Methods

### Strains and culture conditions

*K. brevis* clone SP3 and TXB4 were isolated from a red tide bloom near South Padre Island, Texas in 1999^25^. Two inoculum cultures (one for SP3, one for TXB4) were started at 1200 cells/ml and grown in L1 medium made from seawater collected offshore in the GoM^26^. The seawater was prepared by filtration through a 0.2 um polycap capsule filter (Whatman Inc.), dilution to a salinity of 32 psu with MilliQ water, and autoclaving. The cultures were maintained at an irradiance level of 50 µmol m^−2^ s^−1^ on a 12:12 h light:dark cycle with cool white fluorescent bulbs at 25 °C. When the concentration of cells in each inoculum culture reached about 1500 cells/ml, 60 ml of algal culture was dispensed into each of 18, 125 ml flasks, and maintained under the same environmental conditions. Among these 18 flasks, sets of 3 flasks were designated as “treatment 500”, “treatment 750”, “treatment 1000”, “control 500”, “control 750” and “control 1000”.

### Experimental setup and sampling

Experimental high light intensities of 500, 750 and 1000 µmol m^−2^ s^−1^ were achieved by using two sets of light banks with multiple dimmable small cool white lights (Bozily Aquarium Lights LED). The light intensities were confirmed by LI-192 quantum sensor connected to an LI-1400 DataLogger (LI-COR Biosciences). During the log phase of culture growth, the triplicate flasks of treatment 500 were moved from 50 to 500 µmol m^−2^ s^−1^ at the beginning of a light period (time 0 h), while the 12:12 h light:dark cycle was retained. Meanwhile, the triplicate flasks of control 500 were kept at 50 µmol m^−2^ s^−1^ with the same light:dark cycle as the treatments. Because time zero was the end of the dark phase, the first sampling point for both treatments and controls was at time 0.5 h, followed by subsequent sampling at 6 h and 24.5 h. The experiments for 750 and 1000 µmol m^−2^ s^−1^ were set up in the same way, except that the sets of treatment 750 and treatment 1000 flasks were transferred to their corresponding light conditions. Experiments for these three light treatments were conducted on different but consecutive days (Day 1 and Day 2 for treatment 500; Day 3 and Day 4 for treatment 750; Day 5 and Day 6 for treatment 1000), so a separate control set was used and sampled together with each treatment set.

Although there were differences in starting live cell densities across different treatment sets, all cultures were in log phase throughout the duration of the experiments. Thus, all cultures were supposed to have similar initial physiological status before the introduction of stress. In addition, separated control set were set up and sampled together with their corresponding treatment set, which would be used to compare and reflect the cellular photo-physiological changes induced by the high light exposure.

### Measurement of stress and cell death markers

Three PCD-related parameters were determined by fluorescent labeling as described below to calculate the prevalence of cells showing cell death, ROS and caspase-like activity. An epifluorescent microscope (BX41, Olympus) was used to observe green fluorescence generated by these reagents with a filter that has a band pass excitation wavelength of 450–490 nm and long pass emission of 523 nm. Approximately 50–100 cells were counted and examined, in replicate, for each parameter from each experimental flask, using a Sedgewick Rafter chamber.

#### Cell death detection

SYTOX-green dye (S7020, Invitrogen) was used to examine the integrity of cell membrane^8,27,28^. Compromised cell membrane is considered as an indicator of cell death. This reagent has a high affinity with nucleic acid and can only pass through impaired cell membranes. Working solution of SYTOX-Green (2.5 μl; 100 μM) was mixed with 500 μl of algal culture sample, resulting in a final concentration of 0.5 μM. After 40 min dark incubation at room temperature SYTOX-stained cells were examined. SYTOX percentages were calculated as: the number of cells with green fluorescence/the number of intact cells.

#### ROS production

Carboxy-H_2_DCFDA (C400, Invitrogen) was used to detect intracellular ROS in *K. brevis* cells^9,29–31^. For preparing the stock solution, 2 mg of carboxy-H_2_DCFDA were dissolved in DMSO at a concentration of 10 mM. Before the experiment PBS was used to prepare the working solution by diluting an aliquot of stock solution to 100 μM. Then the working solution was added into the algal sample at a 1:9 ratio, resulting in a 10 μM final concentration of carboxy-H_2_DCFDA. Cells were incubated for 20 min at room temperature in the dark before the examination of green fluorescence resulting from the oxidation of carboxy-H_2_DCFDA. Prevalence of cells with ROS was calculated as the number of cells with green fluorescence/ the number of intact cells.

#### Activity of caspase-like enzymes

Activation of caspase-like enzymes is a distinctive characteristic of PCD. CellEvent in situ caspase 3/7 Green Detection Reagent (C10423, Invitrogen) was used to detect caspase-like activity in *K. brevis* cells^30^. The reagent was diluted by a subsample of *K. brevis* cultures to a final concentration of 10 μM. Observations of stained cells were performed after at least 30 min of room temperature incubation. Percentage of cells with caspase-like activity was calculated as: the number of cells with green fluorescence/intact cell number.

### Live cell density

The densities of total morphologically intact cells were estimated, in duplicate, from each experiment flask via microscope using a Sedgewick Rafter counting chamber after Lugol’s preservation. A morphologically intact cell refers to the cell that retains basic membrane and intracellular structure. Densities of live cells from each flask were calculated as: averaged total morphologically intact cells per ml × (1 − averaged SYTOX proportion).

### Photosynthetic parameters

At each sampling time 2 ml of algal culture were collected from each flask and placed in the dark for 30 min. Fast Repetition Rate fluorometry (FRRf) on a FastTRACKA instrument (Chelsea Technologies Group Ltd.) was used to measure the photosynthetic efficiency of photosystem II (Fv/Fm), where Fm is the maximal fluorescence excited by a saturating pulse of light (> 10,000 µmol m^−2^ s^−1^) in a dark-adapted sample, and Fv is the difference between minimal fluorescence (F_0_; 0.15 µmol m^−2^ s^−1^) and Fm. In addition, Rapid Light Curves (RLC) were also collected by FRRf to derive the potential maximum relative electron transport rate (potential rETRmax, an approximation of the potential maximum rate of electrons pumped through the electron transfer chain; dimensionless) and the efficiency of photosynthesis (alpha, initial slope of the rETR vs. irradiance curve; µmol^−1^ m^2^ s^−1^). RLC measurements were conducted with a stepped actinic irradiation of 10 s duration from 0 to ∼ 1000 µmol m^−2^ s^−1^ (0, 5, 22, 92, 216, 356, 499, 554, 646, 758, 846, 915 and 1019 µmol m^−2^ s^−1^). RLC data were analyzed in R software using the package phytotools. The FRRf was conducted with single turnover (ST) acquisition. An ST sequence is composed of 100 saturation flashes of 1 µs duration with an interval of 1 µs in between. For each acquisition twelve sequences were performed with an interval of 100 ms between each sequence^32^. In order to calculate non-photochemical quenching (NPQ), another 2 mL sample was collected from each flask at each sampling time, and then maximum fluorescence yield of a light-adapted samples (F’m) was measured by FRRf. NPQ was calculated by the following equation:

$${\text{NPQ }} = \, \left( {{\text{Fm }} - {\text{ F}}^{\prime } {\text{m}}} \right)/{\text{F}}^{\prime } {\text{m}}),$$

and if $${\text{F}}^{\prime } {\text{m }} > {\text{Fm then NPQ }} = \, 0$$^33^.

### Statistical analysis

Two-way repeated measures ANOVA (RM-ANOVA) was used to compare cellular parameters between treatments and controls at each sampling time point. The Bonferroni post-hoc test was used to determine significance of the differences. The original α is equal to 0.05. All statistical analysis was performed using SigmaPlot (Version 14.0, Systat Software).

## Results

**Figure 1:**
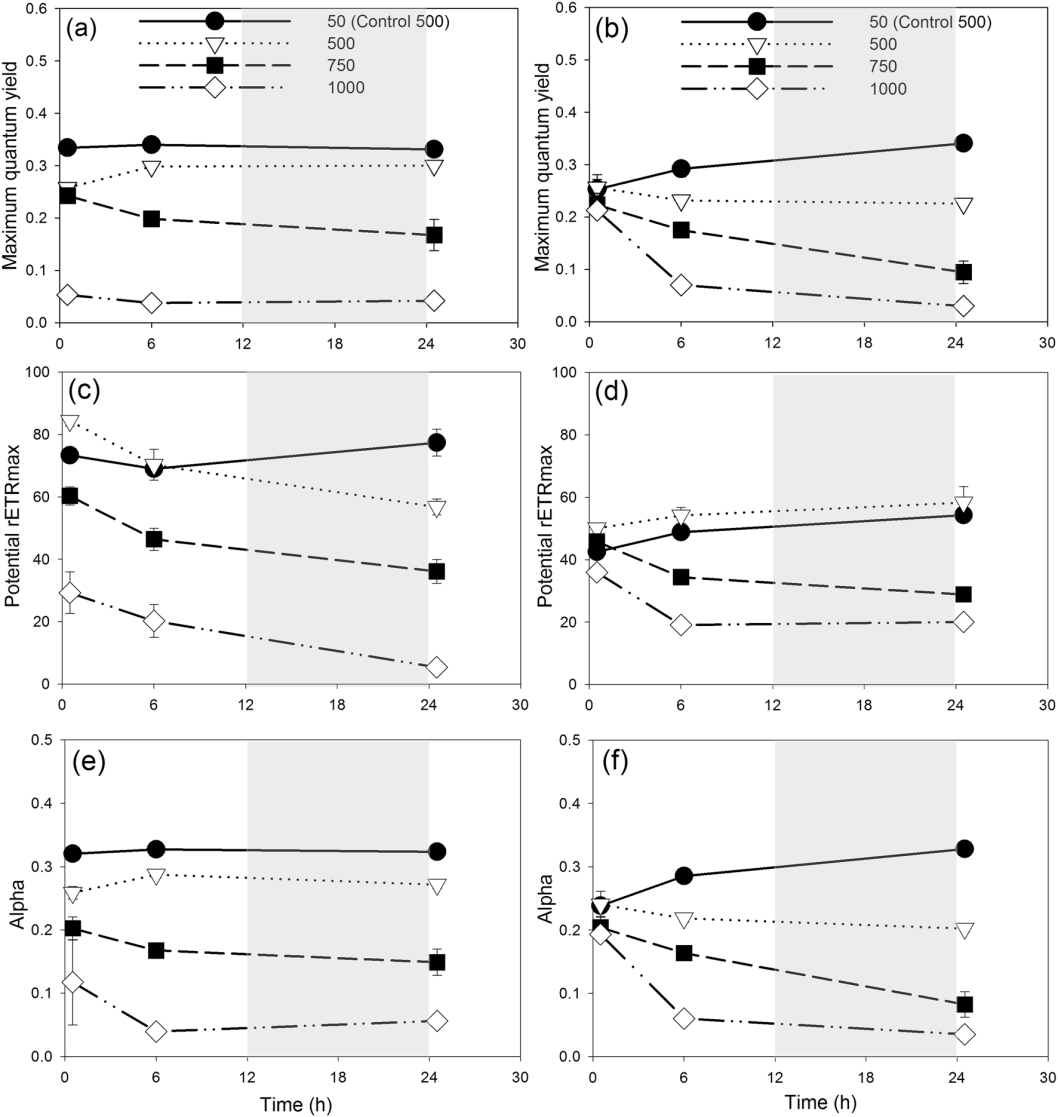
Photosynthetic responses in *K.brevis* strain SP3 (**a**,**c**,**e**) and TXB4 (**b**,**d**,**f**) transferred from 50 to 500, 750 and 1000 µmol m^−2^ s^−1^ and the controls (50 µmol m^−2^ s^−1^). Variations in Fv/Fm (**a**,**b**), potential rETRmax (**c**,**d**) and alpha (**e**,**f**). Gray shading indicates dark phase. Data points show average values of triplicate treatment/control, and error bars show standard deviation of the replicates (n = 3). Note: the expression of photosynthetic parameters across different control set were generally similar. In order to keep the figure readable and uncluttered, among all the control set only “control 500” is shown in the figure.

Photosynthetic function in strain SP3 and TXB4 generally decreased with increasing light intensities (Fig. 1, Supplementary Tables S1, S2, S3). The Fv/Fm in treatments was characterized by a dose-dependent decrease with increasing irradiance and was significantly lower than controls at each time point (*p* < 0.05; Fig. 1a, b) with the exception of TXB4 under 500 µmol m^−2^ s^−1^ at time 0.5 h (*p* = 0.77, Fig. 1b). Maximum photosynthetic rates (rETRmax) and the efficiency of photosynthesis (alpha) showed similar patterns as they both decreased as light stress increased (Fig. 1c, d, e, f). One exception is the rETRmax under 500 µmol m^−2^ s^−1^ (Fig. 1c, d), which did not reduce after stress compared with the controls (strain TXB4) or was only reduced between 6 and 24 h (strain SP3). RLC curves were remarkably depressed after an increase of irradiance by more than 700 µmol m^−2^ s^−1^ in both strains (Supplementary Fig. S1).

**Figure 2:**
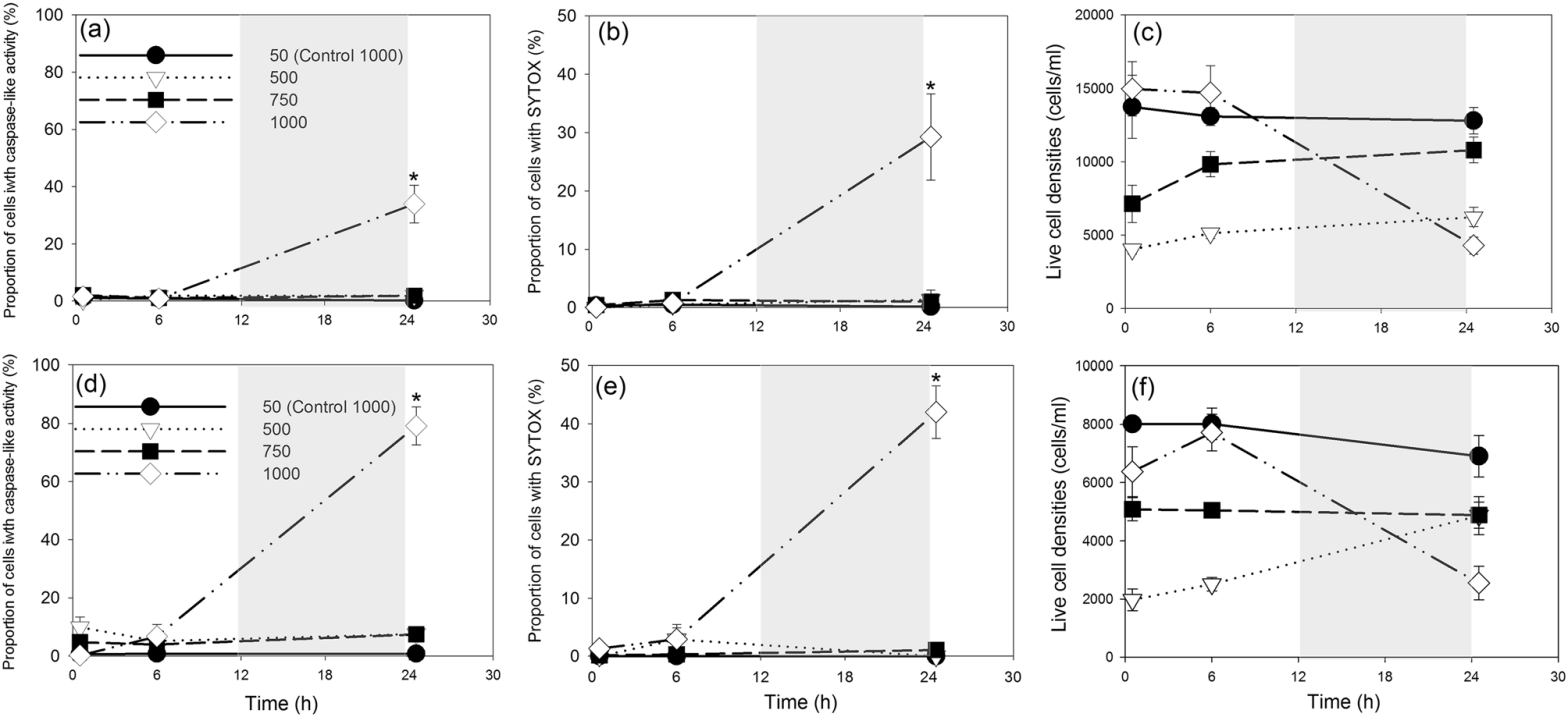
Caspase-like activity (**a**,**d**), cell death (**b**,**e**) and live cell densities (**c**,**f**) in the strain SP3 (**a**,**b**,**c**) and TXB4 (**d**,**e**,**f**) transferred from 50 to 500, 750, and 1000 µmol m^−2^ s^−1^ and the controls (50 µmol m^−2^ s^−1^). Gray shading indicates dark phase. Data points show average values of triplicate treatment/control, and error bars show standard deviation of the replicates (n = 6). Note: the data for control were obtained from “control 1000” cultures. Asterisks indicate a significant difference between treatments and controls (*p* < 0.05).

No significant caspase-like activity and cell death were induced by acute light stress at 500 µmol m^−2^ s^−1^ (10-fold increase) or 750 µmol m^−2^ s^−1^ (15-fold increase) during this 24-h experiment (*p* > 0.05), while self-catalyzed cell death was observed under 1000 µmol m^−2^ s^−1^ at time point 24.5 h (Fig. 2a, b, d, e, Supplementary Tables S4, S5). In treatment 500 of SP3 and TXB4, live cell numbers increased from 4033 ± 314 to 6238 ± 655 cells/ml, and from 1975 ± 368 to 4867 ± 654 cells/ml, respectively (Fig. 2c, f). In treatment 750 of SP3 live cell numbers increased from 7137 ± 1275 to 10,804 ± 876 cells/ml, while in TXB4 live cell numbers varied between 4887 ± 449 and 5087 ± 398 during the 24 h of the experiment (Fig. 2c,f, Supplementary Table S6). Under 1000 µmol m^−2^ s^−1^ significant mortality was observed at time 24.5 h in both strains (Fig. 2). For strain SP3, 33.92 ± 6.59% of cells showed caspase-like activity (Fig. 2a; *p* < 0.05) and SYTOX florescence was detected in 29.27 ± 7.40% of cells (Fig. 2b; *p* < 0.05). For strain TXB4, the prevalence of caspase-like activity reached to 79.09 ± 6.56% with SYTOX percentages of 42.04 ± 4.54% (Fig. 2d,e; *p* < 0.05). Cell loss was severe in both strains, with 60% and 45% of live cells lysed between 6 and 24.5 h in SP3 and TXB4, respectively. In comparison, only background level of caspase-like activity (Fig. 2a,d) and cell death (Fig. 2b,e) were observed during the first 6 h (*p* > 0.05).

**Figure 3:**
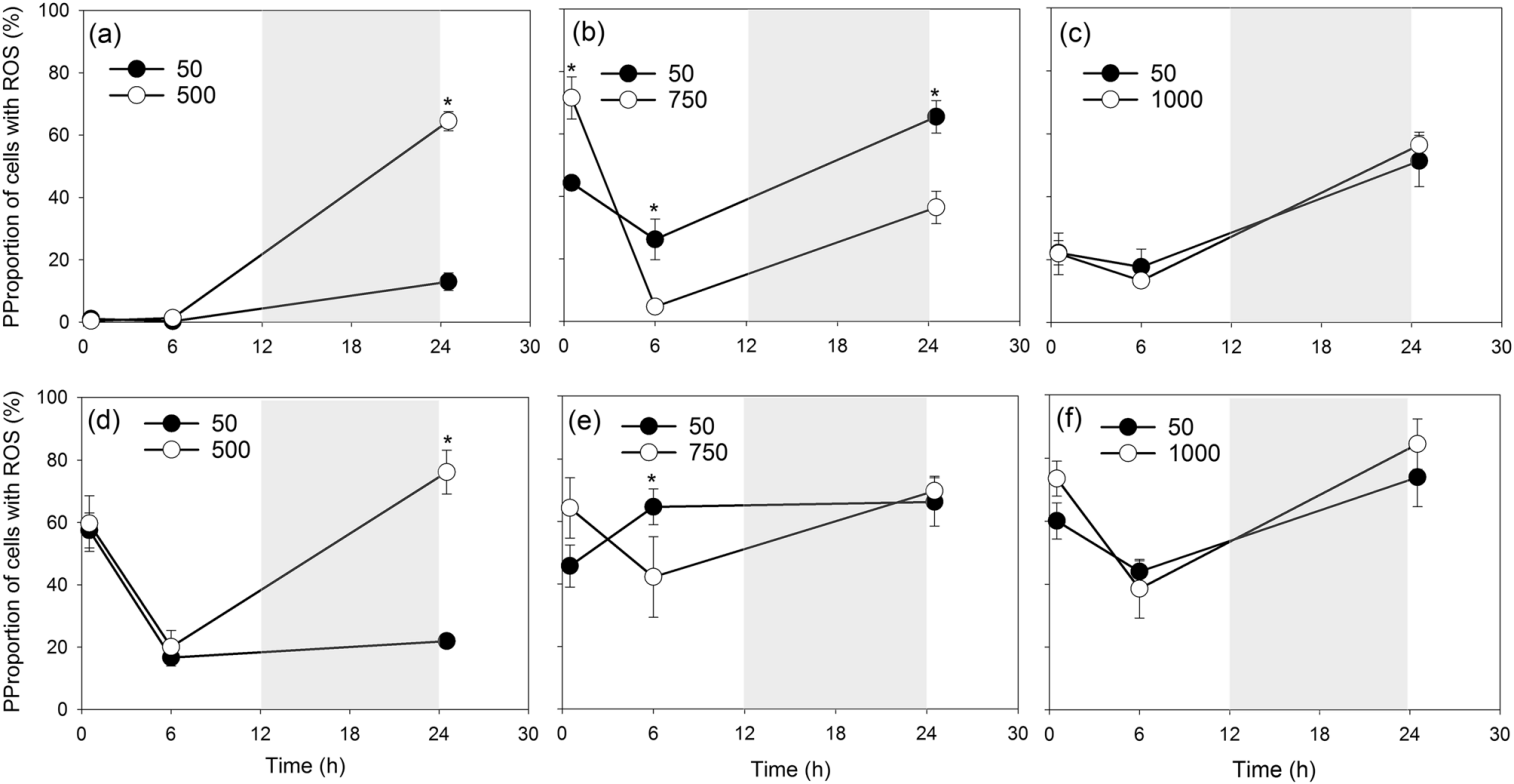
The temporal changing patterns of ROS percentages in the strain SP3 (**a**,**b**,**c**) and TXB4 (**d**,**e**,**f**) transferred from 50 to 500 (**a**,**d**), 750 (**b**,**e**) and 1000 (**c**,**f**) µmol m^−2^ s^−1^ and their corresponding controls (50 µmol m^-2^ s^-1^). Gray shades indicate dark phase. Data points show average values of triplicate treatment/control, and error bars show standard deviation of the replicates (n = 6). Asterisks indicate a significant difference between treatments and controls (*p* < 0.05).

ROS prevalence in *K. brevis* varied under different levels of light stress (Fig. 3). After exposing these two strains to 500 µmol m^−2^ s^−1^, ROS prevalence was not significantly higher than control (*p* > 0.05) until time 24.5 h (Fig. 3a,d). Under 750 µmol m^−2^ s^−1^ the ROS prevalence in both strains showed an immediate increase at time 0.5 h, followed by a significant reduction relative to the controls (SP3: *p* = 0.001; TXB4: *p* = 0.027; Fig. 3b,e). At 1000 µmol m^−2^ s^−1^ the percentages of cells showing an intracellular accumulation of ROS were not significantly different between treatments and controls at any point during the experiment even when there was conspicuous mortality (*p* > 0.05, Fig. 3c,f).

**Figure 4:**
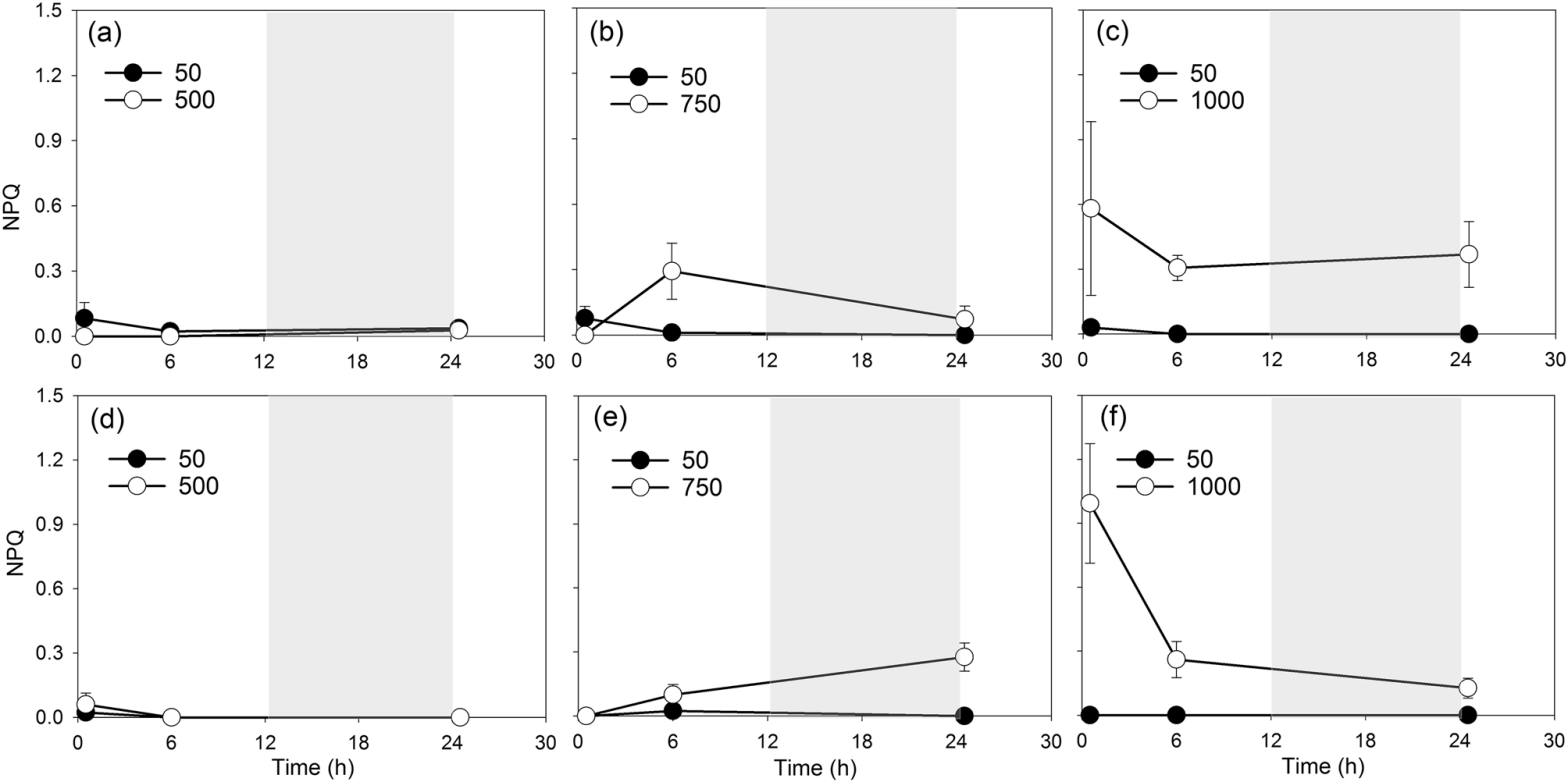
The temporal changing patterns of NPQ in the strain SP3 (**a**,**b**,**c**) and TXB4 (**d**,**e**,**f**) transferred from 50 to 500 (**a**,**d**), 750 (**b**,**e**) and 1000 (**c**,**f**) µmol m^−2^ s^−1^ and their corresponding controls (50 µmol m^−2^ s^−1^). Gray shades indicate dark phase. Data points show average values of triplicate treatment/control, and error bars show standard deviation of the replicates (n = 3).

The intensities and timing of NPQ induction were also different along with increasing light stress (Fig. 4). At 500 µmol m^−2^ s^−1^ NPQ remained very weak at all the sampling time points in both strains even when the increase of ROS prevalence occurred at time 24.5 h (Fig. 4a,d). At 750 µmol m^−2^ s^−1^, NPQ in strain SP3 increased from 0 to 0.29 ± 0.13 between time 0.5 h and time 6 h before dropping to 0.072 ± 0.062 at time 24.5 h (Fig. 4b). In contrast, NPQ in TXB4 increased to a lower level (0.099 ± 0.049) compared to SP3 at time 6 h, but showed increased capacity of NPQ (0.28 ± 0.066) at the beginning of the second light cycle (Fig. 4e). The patterns of NPQ in SP3 and TXB4 are similar at 1000 µmol m^−2^ s^−1^, remaining high at all sampling time points (Fig. 4c,f).

## Discussion

**Figure 5:**
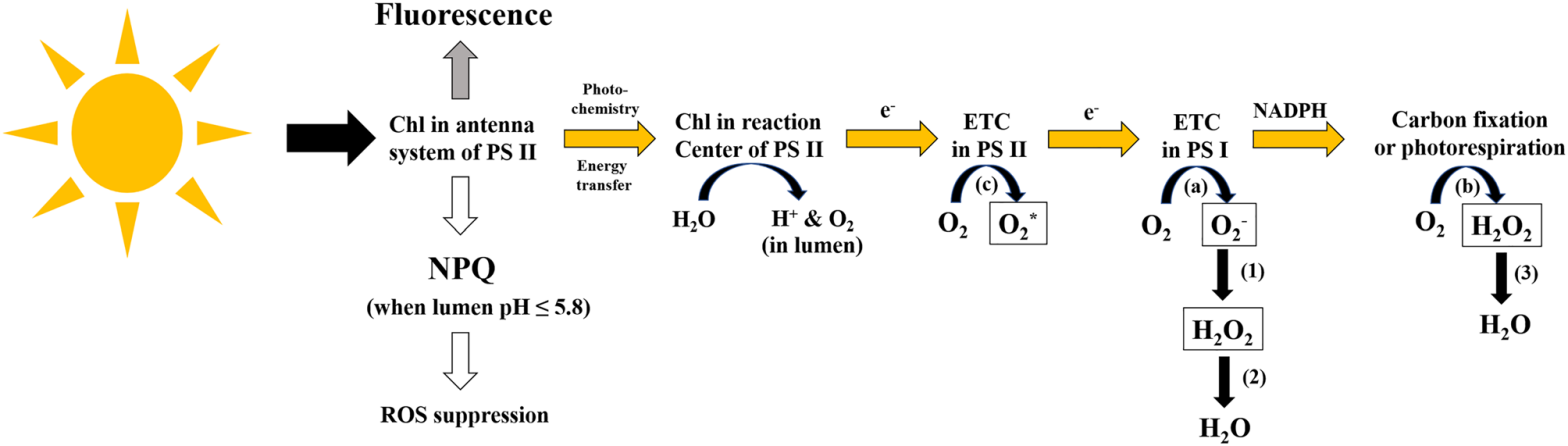
Possible fates of energy absorbed by chlorophylls in microalgae: fluorescence emission, photochemistry and non-photochemical quenching (NPQ). When Chl in the antenna system is excited by absorption of light, some energy can be dissipated via fluorescence emission (gray arrow). Some energy will be transferred to Chl in the reaction center and enter the ETC in the form of electrons (orange arrow), generating oxygen and hydrogen ions by the splitting of H_2_O in the lumen. Under low light, electrons would be transferred through ETC and result in carbon fixation (orange arrows). Under high light, in order to sustain a high level of electron flux, excess electrons would be transferred to oxygen via (**a**) the reduction of oxygen by PSI that produces superoxide (O_2_^−^) and H_2_O_2_, and (**b**) the photorespiratory cascade that generates H_2_O_2_ inside the peroxisome. These ROS species can be converted to H_2_O by (1) superoxide dismutase, (2) ascorbate peroxidase and (3) catalase. Singlet oxygen (O_2_*), a species of ROS, are increasingly generated when the ETC is over-reduced due to high light (**c**). Accumulation of ROS (in black boxes) would trigger PCD pathways. On the other hand, when pH in lumen is lower than 5.8 due to the splitting of H_2_O, NPQ would be activated to dissipate energy of excitation in Chl for the suppression of ROS production (white arrows).

This study investigated cell death processes in response to acute high light stress as well as the dissipation of extra light energy by NPQ in *K .brevis*. Cell death only occurred at the largest light shift (50 to 1000 µmol m^−2^ s^−1^) with concomitant increase of caspase-like activity; no significant mortality or caspase-like enzyme activity were induced at an increase of irradiance up to 700 µmol m^−2^ s^−1^. Oxidative stress and decreased photosynthetic capacities, however, were observed at lower light levels, but did not accompany cell death. The intensity and timing of NPQ induction varied with different light intensities. Light energy absorbed by chlorophyll is dissipated via three main pathways: fluorescence emission, photochemistry and non-photochemical quenching^15,16^ (NPQ; Fig. 5). When a cell is acclimated to its ambient light level, the vast majority of energy is used to fuel photosynthesis by pumping electrons through PS II and PS I, while NPQ and intracellular ROS remain at very low levels^16,22,34^ (Fig. 5). Meanwhile, a small amount of the energy absorbed by chlorophyll is released as fluorescence; the yield of fluorescence is 0.6–3%^16,35^. With increasing light intensity, the flux of electrons in the electron transport chain (ETC) increases. Once the electron flux exceeds the capacity of CO_2_ assimilation, the extra electrons can be shunted to the reduction of oxygen by PSI (Mehler reaction) and the photorespiratory pathway, to protect the ETC against photoinhibition^15^ (Fig. 5). Toxic H_2_O_2_ can be generated by these two processes and can be converted to H_2_O efficiently by antioxidant enzymes^15^ (Fig. 5). However, continuing strong electron flux will eventually lead to overreduction of ETC in PSII and formation of ROS (e.g. singlet oxygen) which may not be scavenged^15,16^ (Fig. 5). Accumulation of ROS can trigger PCD by eliciting caspase-like activity^8,17,36^. In plant cells, another main source of ROS is respiration in mitochondria, but its contribution to cellular ROS load is very low at daytime as a result of efficient scavenging^15,37,38^. Therefore, ROS generated from photosynthesis is the main focus here.

Besides transferring electrons to O_2_, excessive excitation energy in chlorophyll can be dissipated as heat by thermal inactivation of chlorophyll^22^, a process called non-photochemical quenching (NPQ; Fig. 5). NPQ plays an important role in photoprotection, as it accounts for the fate of 75% of absorbed light energy in plants^14,39^. In dinoflagellates, the major type of NPQ is the pH- or energy- dependent component (qE) characterized by rapid induction and relaxation^40^. This rapid type of NPQ has been observed in *K. brevis* under stepped actinic irradiation^41^. The activation of qE pathway is determined by lumen pH which is affected by the splitting of H_2_O in light reaction^22^ (2H_2_O → 4H^+^  + O_2_ + 4e^−^; Fig. 5). In order to gain insights into the effects of ROS production and NPQ-driven ROS inhibition on determining cell death under high light in *K. brevis,* we compared ROS prevalence and NPQ expression to understand their interrelationships under different levels of light stress.

### NPQ-ROS regulation processes under different levels of high light

#### Acute light increase to 500 µmol m^−2^ s^−1^

At 500 µmol m^−2^ s^−1^ photoinhibition appeared to be cumulative, as electron transport capacity (rETRmax) did not show immediate decreases, and significantly higher ROS generation only occurred after at least 6 h of exposure. These observations indicated that *K. brevis* strains were able to manage this level of acute light stress for at least 6 h, but by 24.5 h they could not resist the accumulation of oxidative stress especially for the strain SP3, as its ETC could not keep up with the high flow of energy. However, 500 µmol m^−2^ s^−1^ irradiance was insufficient to induce significant NPQ (SP3: 0 to 0.027 ± 0.012; TXB4: 0 to 0.060 ± 0.052) in *K. brevis*. This is similar to the NPQ of 0.1 and 0.05 triggered in other *K. brevis* strains by 363 µmol m^−2^ s^−1^ irradiance in Cassell et al. (2015)^41^. The sensitivity of NPQ activation can be highly variable among dinoflagellate species. For example, low-light adapted *Karlodinium veneficum* shows a strong NPQ induction of greater than 2.5 after light shock to 300 µmol m^−2^ s^−1^ for ten mins^40^. Moreover, since the culture responses were only examined for the first 24 h after exposure, it is unknown if NPQ would be activated later to balance the observed oxidative burst.

#### Acute light increase to 750 µmol m^−2^ s^−1^

Under acute stress of 750 µmol m^−2^ s^−1^ we observed immediate and sustained reduction in photosynthetic parameters. ROS prevalence increased within the first 30 min, indicating that the light shock rapidly overwhelmed the capacity of some cells to channel the additional light energy. However, intra-specific variation does exist, as SP3 expressed a stronger acute ROS prevalence than TXB4 compared with the controls. High light-induced ROS have been reported in microalgae including diatoms and cyanobacteria. In the diatom *Thalassiosira weissflogii* rapid and intensive H_2_O_2_ production was detected with changing light in the laboratory environment^42^ (150–500 µmol m^−2^ s^−1^), while natural high light remarkably enhances the production of ROS in cyanobacteria *Trichodesmium*^43^. Although NPQ activity was not induced at 30 min, it was observed at time 6 h and time 24.5 h. For SP3, induction of NPQ at time 6 h was accompanied by a disappearance of ROS staining, suggesting that excess energy was diverted into the NPQ pathway and away from the damaged ETC^15,16,44^. After the dark period, in SP3 we saw a repeat of the ROS/NPQ pattern from the day before: ROS prevalence spikes without strong induction of NPQ, which presumably relaxed during the preceding 12 h of dark. In contrast, smaller increase of NPQ capacity may be associated with the smaller decline of ROS prevalence at time 6 h in TXB4, while an intensive induction of NPQ was observed after the dark period, showing different timing to regulate oxidative stress via NPQ in different *K. brevis* strains.

A balancing relationship between NPQ and ROS may exist in *K. brevis*. Cassell et al. (2015) reported that a strain which was deficient in NPQ produced ROS at twice the rate of the strain with high NPQ activity^41^. Previous research has also suggested that conversion of the xanthophyll cycle in NPQ activation can offer significant protection under partially cloudy and sunny days by down-regulating photosynthesis in *K. brevis*^23^. Therefore, photoprotection regulated by NPQ may play a key role in cellular survival in *K. brevis* under sudden light increase. This is evident from the increase in cell densities at time 6 h in SP3, as no significant mortality occurred despite the clear impacts on photosynthetic function. Fluctuation of ROS prevalence in the control culture was also observed, but this corresponds to the diel variation of ROS metabolism described in Gao & Erdner (2021)^45^.

#### Acute light increase to 1000 µmol m^−2^ s^−1^

Cell death was observed under 1000 µmol m^−2^ s^−1^, but rapid induction of the NPQ pathway may have delayed the mortality for a limited period of time. Immediately after the transfer to 1000 µmol m^−2^ s^−1^, photochemistry was reduced by photoinhibition and/or photodamage, as shown by severely or moderately depressed photosynthetic parameters. NPQ was also markedly induced within 30 min of exposure. This induction of NPQ likely reduced the potential for ROS production, thereby delaying the activation of the PCD cascade for at least six hours. Rapid NPQ activation triggered by intensive acute light stress (over 1000 µmol m^−2^ s^−1^) has been observed in rice, diatoms, green algae and cyanobacteria^46,47^. The strain TXB4 experienced a weaker acute depression of photosynthetic performance which may be linked to a higher percentage of light energy dissipating by heat at time 0.5 h. However, Fv/Fm continued to decrease along with the decline of NPQ from time 0.5 h to time 6 h, suggesting that photoinhibition was increasing while photoprotection was weakening. The 1000 µmol m^−2^ s^−1^ irradiance eventually overwhelmed both *Karenia* strains and most of the cells died over the next 18 h as a result of accumulation of photodamage that caused break-down of photosynthetic apparatus. The high prevalence of STYOX staining and caspase-like activity at time 24.5 h indicate autocatalytic cell death. ROS prevalence increased from time 6 h to time 24.5 h in the treatments, likely because there were far fewer live cells. Dead cells have permeable membranes which would not retain small intra-cellular ROS molecules, so ROS detection may be restricted to the few remaining live cells.

### Cell death and tolerance under high light stress in *K. brevis*

Significant cell death was only observed at the highest light levels in *K. brevis*, and it was a self-catalytic process. Although no research on high-light-induced PCD has been reported from dinoflagellates, a cell death pathway with PCD features was reported in cyanobacteria species under high light. Berman-Frank et al. (2004) studied the demise of the marine cyanobacterium *Trichodesmium* in response to 450 µmol m^−2^ s^−1^ light and found that within 3 h of light exposure a large proportion of cells showed low levels of DEVD cleavage (a substrate for caspase), while extended exposure (> 7 h) caused stronger DEVD cleavage and death in cells^48^. Our research is the first report of high-light-induced PCD in a dinoflagellate, revealing the effects of short-term light shock on the viability of *K. brevis* populations.

Our results support the hypothesis that *K. brevis* is a high-light-tolerant species^20^. No significant cell death and caspase-like activity were observed after light shift from 50 to 500 or 750 µmol m^−2^ s^−1^, instead the cell densities maintained or continued to increase. The cells remained viable despite severe depression of photosynthetic capacities under acute light stress. In a previous study that investigated physiological responses of *K. brevis* transferred from 150 to 825 µmol m^−2^ s^−1^ for 4–8 h, there was no difference in viability even though Fv/Fm declined significantly^19^. Tilney et al. (2019) reported dose-dependent decreases of Fv/Fm without growth inhibition from 43 to 1340 µmol m^−2^ s^−1^ in *K. brevis*, leading to the idea that PSII photoinactivation may not affect photosynthesis to a level that would remarkably suppress growth^20^. In addition, *K. brevis* are apparently insensitive to UV in the field, because photosynthetic responses between “PAR-only” and “PAR + UV” treatments from natural sunlight are very similar, and the level of photoinhibition due to high PAR irradiance is significantly higher than the level of inhibition from UV^23^. These characteristics described above may be beneficial for the formation and development of *K. brevis* blooms in GoM, because swimming behavior and upwelling can keep algal cells at sea surface where the noon incident radiation can be as high as 1200 µmol m^−2^ s^−1^^4,23^.

While cell death of *K. brevis* did occur at 1000 µmol m^−2^ s^−1^, it happened only after at least 6 h of exposure. In this case, dose-dependent NPQ expression may have played a key role in preventing or delaying the occurrence of cell death by mitigating ROS production that could trigger the PCD cascade. The timing of NPQ induction varied by light intensity and exposure time, and it was preceded by an increase in ROS prevalence. At the 500 µmol m^−2^ s^−1^ exposure level, significant ROS prevalence was only detected at the last time point in the absence of NPQ induction. At the 750 µmol m^−2^ s^−1^ level, an immediate increase in ROS was followed by a later induction of NPQ, decrease in ROS, and no evidence of cell death. At the highest light exposure, NPQ induction was immediate, and widespread cell mortality occurred after 6 h exposure. In the field, different intensities and timing of NPQ expression may help *K. brevis* cope with natural fluctuations of light intensity, maintaining the viability and development of the population. On the other hand, the occurrence of significant PCD and cell lysis after 6 h of 1000 µmol m^−2^ s^−1^ also revealed the limitations of photoprotection from NPQ as well as the potential effects of extreme high light on the sustainability of *K. brevis* populations.

## Conclusions

This is the first report of high-light induced PCD in a dinoflagellate. Cell death in *K. brevis* only occurred at the largest light shift (50 to 1000 µmol m^−2^ s^−1^) and was associated with an increase of caspase-like activity. This research supports strong light-tolerance in *K. brevis*, as no significant mortality and caspase-like activity were observed under 700 µmol m^−2^ s^−1^ increase of irradiance. However, depressed photosynthetic capacities and oxidative stress were apparent, but did not necessarily lead to cell death. NPQ exhibited dose-dependent activation with increasing light stress and duration; it enables cells to resist or delay PCD by mitigating ROS production under certain levels of acute light stress. These results highlight the important role of the balance between ROS generation and NPQ activation on determining cell fates in *Karenia* under acute irradiance stress. They also provide insights into potential survival strategies as well as mechanisms of bloom decline under the changeable light environment in the field.

## Supplementary Information

Supplementary Information.

## Data Availability

All data generated or analysed during this study are included in this article and supplementary tables.
