# Supplementary material for: Cell death responses to acute high light mediated by non-photochemical quenching in the dinoflagellate Karenia brevis
Source: Sci Rep. 2022 Aug 18;12:14081. doi: 10.1038/s41598-022-18056-4 (PMC9388518; doi:10.1038/s41598-022-18056-4)
Supplement: Supplementary file 1 — Supplementary Information. [file 41598_2022_18056_MOESM1_ESM.pdf]

Cell Death Responses to Acute High Light Mediated by Non-Photochemical Quenching in the dinoflagellate *Karenia brevis*

Supplementary Tables & Figures

Yida Gao[1*a], Deana L. Erdner[1],

[1]Marine Science Institute, The University of Texas at Austin, Port Aransas, Texas, USA

*** Correspondence:**
Yida Gao
yida.gao@utexas.edu
[a] Current address: Florida Fish and Wildlife Conservation Commission, Fish and Wildlife Research Institute, 100 8th Avenue SE, St. Petersburg, Florida 33701, USA

Supplementary Table S1. Averaged Fv/Fm and standard error from the strain SP3 and TXB4 transferred from 50 to 500, 750, and 1000 µmol m^{-2} s^{-1} and their corresponding controls (50 µmol m^{-2} s^{-1}). P-value between treatments and controls from RM-ANOVA analysis are also listed (n =3).

SP3

	**500**			**750**			**1000**		
	Treatment	Control	P value	Treatment	Control	P value	Treatment	Control	P value
Time 0.5	0.26 ± 0.01	0.33 ± 0.00	$p < 0.05$	0.24 ± 0.01	0.38 ± 0.01	$p < 0.05$	0.05 ± 0.00	0.38 ± 0.01	$p < 0.05$
Time 6	0.30 ± 0.01	0.34 ± 0.00	$p < 0.05$	0.20 ± 0.01	0.37 ± 0.01	$p < 0.05$	0.04 ± 0.01	0.40 ± 0.00	$p < 0.05$
Time 24.5	0.30 ± 0.01	0.33 ± 0.01	$p < 0.05$	0.17 ± 0.03	0.34 ± 0.01	$p < 0.05$	0.04 ± 0.01	0.36 ± 0.01	$p < 0.05$

TXB4

	**500**			**750**			**1000**		
	Treatment	Control	P value	Treatment	Control	P value	Treatment	Control	P value
Time 0.5	0.26 ± 0.02	0.25 ± 0.02	$p > 0.05$	0.22 ± 0.02	0.38 ± 0.00	$p < 0.05$	0.21 ± 0.01	0.37 ± 0.00	$p < 0.05$
Time 6	0.23 ± 0.01	0.29 ± 0.02	$p < 0.05$	0.18 ± 0.01	0.38 ± 0.00	$p < 0.05$	0.07 ± 0.01	0.36 ± 0.00	$p < 0.05$
Time 24.5	0.23 ± 0.00	0.34 ± 0.00	$p < 0.05$	0.09 ± 0.02	0.37 ± 0.00	$p < 0.05$	0.03 ± 0.00	0.37 ± 0.00	$p < 0.05$

Supplementary Table S2. Averaged potential rETRmax and standard error from the strain SP3 and TXB4 transferred from 50 to 500, 750, and 1000 µmol m^{-2} s^{-1} and their corresponding controls (50 µmol m^{-2} s^{-1}). P-value between treatments and controls from RM-ANOVA analysis are also listed (n = 3).

SP3

	**500**			**750**			**1000**		
	Treatment	Control	P value	Treatment	Control	P value	Treatment	Control	P value
Time 0.5	84.33 ± 1.75	73.28 ± 0.16	p > 0.05	60.31 ± 2.89	81.32 ± 3.35	p < 0.05	29.30 ± 6.69	70.14 ± 2.08	p < 0.05
Time 6	70.26 ± 4.95	69.01 ± 1.46	p > 0.05	46.41 ± 3.49	79.37 ± 1.86	p < 0.05	20.26 ± 5.26	85.67 ± 8.96	p < 0.05
Time 24.5	56.80 ± 2.57	77.35 ± 4.30	p < 0.05	36.13 ± 3.81	65.91 ± 3.47	p < 0.05	5.42 ± 0.00	76.83 ± 1.19	p < 0.05

TXB4

	**500**			**750**			**1000**		
	Treatment	Control	P value	Treatment	Control	P value	Treatment	Control	P value
Time 0.5	50.13 ± 1.19	42.50 ± 4.62	p > 0.05	45.88 ± 0.96	59.08 ± 1.57	p < 0.05	35.93 ± 0.00	48.21 ± 1.87	p < 0.05
Time 6	54.22 ± 2.56	48.84 ± 0.31	p > 0.05	34.39 ± 2.29	57.39 ± 0.70	p < 0.05	19.12 ± 1.66	50.19 ± 0.68	p < 0.05
Time 24.5	58.31 ± 5.08	54.25 ± 1.82	p > 0.05	28.88 ± 2.31	59.73 ± 0.84	p < 0.05	20.10 ± 1.41	55.60 ± 1.69	p < 0.05

Supplementary Table S3. Averaged alpha and standard error from the strain SP3 and TXB4 transferred from 50 to 500, 750, and 1000 µmol m^{-2} s^{-1} and their corresponding controls (50 µmol m^{-2} s^{-1}). P-value between treatments and controls from RM-ANOVA analysis are also listed (n = 3).

SP3

	**500**			**750**			**1000**		
	Treatment	Control	P value	Treatment	Control	P value	Treatment	Control	P value
Time 0.5	0.26 ± 0.01	0.32 ± 0.01	p < 0.05	0.20 ± 0.02	0.38 ± 0.01	p < 0.05	0.12 ± 0.07	0.36 ± 0.01	p < 0.05
Time 6	0.29 ± 0.01	0.33 ± 0.00	p < 0.05	0.17 ± 0.01	0.37 ± 0.01	p < 0.05	0.04 ± 0.01	0.39 ± 0.00	p < 0.05
Time 24.5	0.27 ± 0.01	0.32 ± 0.00	p < 0.05	0.15 ± 0.02	0.33 ± 0.01	p < 0.05	0.06 ± 0.00	0.34 ± 0.00	p < 0.05

TXB4

	**500**			**750**			**1000**		
	Treatment	Control	P value	Treatment	Control	P value	Treatment	Control	P value
Time 0.5	0.24 ± 0.02	0.24 ± 0.01	p > 0.05	0.20 ± 0.02	0.34 ± 0.01	p < 0.05	0.19 ± 0.01	0.36 ± 0.00	p < 0.05
Time 6	0.22 ± 0.01	0.29 ± 0.01	p < 0.05	0.16 ± 0.01	0.36 ± 0.00	p < 0.05	0.06 ± 0.01	0.34 ± 0.00	p < 0.05
Time 24.5	0.20 ± 0.01	0.33 ± 0.01	p < 0.05	0.08 ± 0.02	0.35 ± 0.01	p < 0.05	0.04 ± 0.00	0.35 ± 0.00	p < 0.05

Supplementary Figure S1. Rapid light curves (RLC) from *K .brevis* strain SP3 (a, c, e) and TXB4 (b, d, f) exposed to 50 (controls), 500, 750 and 1000 µmol m^{-2} s^{-1} at time 24.5h. RLC under 500 µmol m^{-2} s^{-1} (a, b), 750 µmol m^{-2} s^{-1} (c, d), 1000 µmol m^{-2} s^{-1} (e, f). Error bars show standard deviation of the replicates (n = 3).

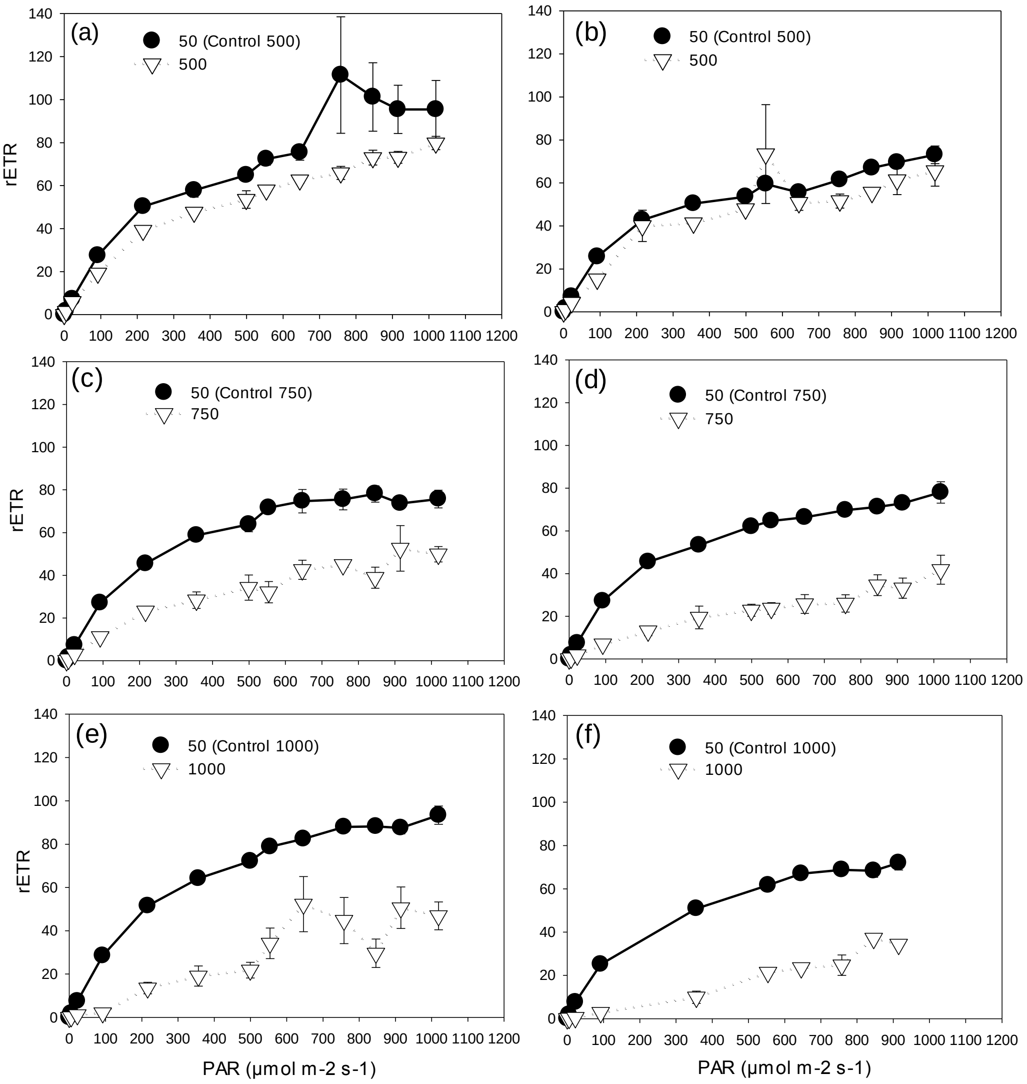

Supplementary Table S4. Averaged caspase-like activity and standard error (n = 6) from the strain SP3 and TXB4 transferred from 50 to 500, 750, and 1000 µmol m^{-2} s^{-1} and their corresponding controls (50 µmol m^{-2} s^{-1}).

SP3

	**500**		**750**		**1000**	
	Treatment	Control	Treatment	Control	Treatment	Control
Time 0.5	0.72 ± 0.40	0.44 ± 0.35	2.09 ± 1.96	3.52 ± 2.82	1.67 ± 1.30	1.14 ± 0.68
Time 6	0.45 ± 035	1.85 ± 0.84	1.06 ± 0.44	0.73 ± 0.58	0.89 ± 0.54	0.92 ± 0.80
Time 24.5	0.53 ± 0.42	1.93 ± 0.79	1.87 ± 0.47	0.28 ± 0.34	33.93 ± 6.59	0.21 ± 0.26

TXB4

	**500**		**750**		**1000**	
	Treatment	Control	Treatment	Control	Treatment	Control
Time 0.5	9.90 ± 3.59	12.96 ± 2.43	4.75 ± 1.92	6.70 ± 1.71	0.27 ± 0.33	0.62 ± 0.35
Time 6	5.19 ± 2.99	1.81 ± 1.41	3.98 ± 1.33	3.68 ± 1.46	6.76 ± 4.13	0.80 ± 0.66
Time 24.5	7.62 ± 1.69	4.54 ± 1.43	7.48 ± 2.60	6.05 ± 1.30	79.09 ± 6.56	0.79 ± 0.66

Supplementary Table S5. Averaged prevalence of cell death (SYTOX percentages) and standard error (n = 6) from the strain SP3 and TXB4 transferred from 50 to 500, 750, and 1000 µmol m^{-2} s^{-1} and their corresponding controls (50 µmol m^{-2} s^{-1}).

SP3

	**500**		**750**		**1000**	
	Treatment	Control	Treatment	Control	Treatment	Control
Time 0.5	3.68 ± 1.69	0.00 ± 0.00	0.43 ± 0.33	0.00 ± 0.00	0.00 ± 0.00	0.33 ± 0.41
Time 6	1.42 ± 0.91	0.56 ± 0.68	1.30 ± 0.88	1.34 ± 0.75	0.76 ± 0.61	0.51 ± 0.42
Time 24.5	0.26 ± 0.32	1.33 ± 1.63	1.03 ± 0.42	0.40 ± 0.49	29.27 ± 7.40	0.17 ± 0.21

TXB4

	**500**		**750**		**1000**	
	Treatment	Control	Treatment	Control	Treatment	Control
Time 0.5	0.00 ± 0.00	1.04 ± 1.28	0.18 ± 0.22	2.39 ± 1.38	1.34 ± 0.78	0.00 ± 0.00
Time 6	2.92 ± 2.55	1.96 ± 1.52	0.34 ± 0.42	1.23 ± 0.96	2.94 ± 1.91	0.00 ± 0.00
Time 24.5	0.00 ± 0.00	0.00 ± 0.00	1.10 ± 0.90	0.00 ± 0.00	42.04 ± 4.54	0.00 ± 0.00

Supplementary Table S6. Averaged live cell densities and standard error (n = 6) from the strain SP3 and TXB4 transferred from 50 to 500, 750, and 1000 µmol m^{-2} s^{-1} and their corresponding controls (50 µmol m^{-2} s^{-1}).

SP3

	**500**		**750**		**1000**	
	Treatment	Control	Treatment	Control	Treatment	Control
Time 0.5	4033 ± 314	4150 ± 109	7137 ± 1276	11233 ± 1217	14975 ± 1846	13746 ± 2148
Time 6	5131 ± 322	7124 ± 588	9820 ± 791	11577 ± 1623	14713 ± 1840	13087 ± 622
Time 24.5	6238 ± 656	7922 ± 440	10804 ± 877	11888 ± 333	4276 ± 542	12801 ± 907

TXB4

	**500**		**750**		**1000**	
	Treatment	Control	Treatment	Control	Treatment	Control
Time 0.5	1975 ± 368	986 ± 113	5087 ± 398	5147 ± 323	6377 ± 855	8010 ± 240
Time 6	2515 ± 233	2685 ± 190	5051 ± 263	6713 ± 431	7718 ± 633	8017 ± 542
Time 24.5	4867 ± 654	2200 ± 389	4887 ± 449	3817 ± 261	2552 ± 576	6908 ± 709
